# Supplementary material for: Knowledge graph-guided multiple sclerosis identification and therapeutic trend analysis: Real-world evidence from two large healthcare systems
Source: PLOS Digit Health. 2026 Aug 28;5(8):e0001554. doi: 10.1371/journal.pdig.0001554 (PMC13524337; doi:10.1371/journal.pdig.0001554)
Supplement: Supplementary file 1 [file pdig.0001554.s001.pdf]

S1 Table. MS phenotyping model (KOMAP) features and coefficients

Feature[1]	Description	UPMC	MGB
		Coefficient	Coefficient
C0004093	Asthenia	0.006	0.000
C0004268	Attention	0.000	-0.013
C0010957	Tissue damage	0.000	0.000
C0011303	Demyelinating Diseases	0.003	0.020
C0011304	Demyelination	0.005	0.019
C0011570	Mental Depression	0.000	0.000
C0011581	Depressive disorder	-0.002	0.000
C0013415	Dysthymic Disorder	0.000	0.000
C0026769	Multiple Sclerosis	0.573	0.545
C0026838	Muscle Spasticity	0.023	0.038
C0029134	Optic Neuritis	0.009	0.023
C0035020	Relapse	0.031	0.054
C0037179	Unmarried person	-0.001	-0.009
C0041696	Unipolar Depression	0.000	0.000
C0066677	modafinil	0.000	0.000
C0069426	Oligoclonal Bands (protein)	0.000	0.000
C0087136	Unmarried	-0.001	-0.009
C0159028	General symptom	0.000	0.000
C0163712	Relate - vinyl resin	-0.002	-0.009
C0202205	Oligoclonal protein measurement	0.000	0.000
C0231170	Disability	0.001	0.000
C0289884	glatiramer acetate	0.000	0.000
C0344315	Depressed mood	0.000	0.000
C0349217	Depressive episode, unspecified	0.000	0.000
C0423551	Sensory symptoms	0.000	0.000
C0528175	Copaxone	0.040	0.065
C0682148	Disability status	0.000	0.000
C0722882	Provigil	0.009	0.016
C0751967	Multiple Sclerosis, Relapsing-Remitting	0.018	0.035
C0878575	Peripheral demyelination	0.005	0.020
C1172734	natalizumab	0.000	0.000
C1269683	Major Depressive Disorder	0.000	0.000
C1457887	Symptoms	0.000	0.000
C1529600	Tysabri	0.035	0.014
C1579931	Depressed - symptom	0.000	0.000
C1857639	Dysmyelination	0.000	0.000
C2362914	Clinical depression	0.000	0.000
C2752009	White matter lesion	0.004	0.035
C3540014	Central Nervous System Diagnostic Radiopharmaceuticals	0.000	0.000
C3714552	Weakness	0.004	0.000
C5202921	RECIL PD	0.000	0.000
C5203119	Intensity and Distress 5	-0.014	-0.008
PheCode:296	mood disorders	0.000	0.000
PheCode:296.2	depression	0.000	0.000
PheCode:296.22	major depressive disorder	0.000	0.000
PheCode:300	anxiety disorders	-0.004	0.000
PheCode:334	degenerative disease of the spinal cord	0.000	0.000
PheCode:335	multiple sclerosis	0.584	0.648
PheCode:341	other demyelinating diseases of central nervous system	0.000	0.000
PheCode:350.1	abnormal involuntary movements	0.000	0.000
PheCode:377.3	optic neuritis/neuropathy	0.000	0.000
PheCode:596.5	functional disorders of bladder	0.023	0.016
RXNORM:1012892	fingolimod	0.003	0.000
RXNORM:1292	baclofen	0.019	0.014
RXNORM:1310520	teriflunomide	0.000	0.000
RXNORM:1373478	dimethyl fumarate	0.027	0.012
RXNORM:214582	glatiramer	0.011	0.002
RXNORM:30125	modafinil	0.010	0.004
RXNORM:354770	natalizumab	0.024	0.000
RXNORM:72257	interferon beta-1b	0.000	0.000
RXNORM:75917	interferon beta-1a	0.035	0.009
RXNORM:897018	dalfampridine	0.011	0.000
utls	Healthcare Utility	-0.542	-0.200

[1] Feature: We used the Online Narrative and Codified feature Search engine (*i.e.,* ONCE) to select 20 codified and 47 NLP-derived features relevant for MS.
