# Supplementary material for: Knowledge graph-guided multiple sclerosis identification and therapeutic trend analysis: Real-world evidence from two large healthcare systems
Source: PLOS Digit Health. 2026 Aug 28;5(8):e0001554. doi: 10.1371/journal.pdig.0001554 (PMC13524337; doi:10.1371/journal.pdig.0001554)
Supplement: Supplementary file 2 [file pdig.0001554.s002.pdf]

S2 Table. MS phenotyping model (KOMAP) performance with different feature sets.

		UPMC				MGB			
		Main PheCode[1]	Main CUI[2]	Codified[3]	Both[4]	Main PheCode[1]	Main CUI[2]	Codified[3]	Both[4]
AUROC		0.854	0.798	0.912	0.922	0.942	0.983	0.974	0.994
AUPROC		0.941	0.898	0.963	0.966	0.831	0.839	0.879	0.94
0.970 Specificity	Sensitivity	0.502	0.209	0.631	0.674	0.605	0.747	0.609	0.957
	PPV	0.976	0.944	0.981	0.982	0.876	0.897	0.868	0.912
	NPV	0.436	0.327	0.510	0.541	0.881	0.921	0.884	0.986
0.950 Specificity	Sensitivity	0.545	0.268	0.711	0.698	0.637	0.82	0.645	0.957
	PPV	0.967	0.935	0.975	0.974	0.876	0.847	0.808	0.861
	NPV	0.454	0.341	0.567	0.557	0.888	0.941	0.892	0.985
0.900 Specificity	Sensitivity	0.662	0.425	0.791	0.794	0.716	0.921	1.000	1.000
	PPV	0.951	0.92	0.952	0.953	0.732	0.761	0.764	0.764
	NPV	0.518	0.385	0.630	0.634	0.907	0.971	1.000	1.000

[1] Main MS PheCode: KOMAP with log(1+ aggregated MS PheCode counts).
[2] Main MS CUI: KOMAP with log(1+ aggregated MS CUI counts).
[3] Codified: KOMAP with log(ICD+1) as surrogate plus 20 codified features selected by ONCE.
[4] Both: KOMAP with log(ICD+1) and log(NLP+1) as surrogate plus 20 ONCE-selected codified features plus 47 ONCE-selected NLP-derived narrative features.

ONCE, Online Narrative and Codified feature Search engine powered by the Online Narrative and Codified feature Search engine
