# Supplementary material for: Knowledge graph-guided multiple sclerosis identification and therapeutic trend analysis: Real-world evidence from two large healthcare systems
Source: PLOS Digit Health. 2026 Aug 28;5(8):e0001554. doi: 10.1371/journal.pdig.0001554 (PMC13524337; doi:10.1371/journal.pdig.0001554)
Supplement: Supplementary file 3 [file pdig.0001554.s003.pdf]

S3 Table. Trends of individual MS-related DMT between 2004 and 2022.

DMT Class	Follow-up Period	Trend[1]	Change Point	Before Change Point		After Change Point	
				β (95% CI)	p	β (95% CI)	p
**Common Prescribed DMTs**							
**dimethyl fumarate**	2013-2022	D-NA	2020	-0.027 (-0.042, -0.012)	0.004	-0.091 (-0.229, 0.046)	0.155
**fingolimod**	2010-2022	NA-D	2012	0.022 (-0.010, 0.053)	0.156	-0.026 (-0.042, -0.011)	0.004
**glatiramer acetate**	2000-2022	I-D	2011	0.22 (0.012, 0.032)	<0.001	-0.013 (-0.025, -0.002)	0.026
**interferon beta-1a**	2000-2022	NA-D	2012	-0.006 (-0.016, 0.005)	0.262	-0.022 (-0.037, -0.007)	0.007
**interferon beta-1b**	2000-2022	NA-D	2002	0.036 (-0.117, 0.190)	0.625	-0.013 (-0.021, -0.004)	0.005
**natalizumab**	2004-2022	I-D	2011	0.063 (0.050, 0.077)	<0.001	-0.027 (-0.035, -0.019)	<0.001
**ocrelizumab**	2017-2022	NA-I	2018	0.010 (-0.039, 0.059)	0.465	0.020 (0.004, 0.035)	0.032
**rituximab**	2022-2022	I-I	2008	0.045 (0.016, 0.074)	0.004	0.012 (0.002, 0.023)	0.020
**teriflunomide**	2012-2022	D	NA	-0.017 (-0.027, -0.006)	0.006	-0.017 (-0.027, -0.006)	0.006
**methylprednisolone**	2000-2022	D-I	2014	-0.023 (-0.029, -0.016)	<0.001	0.029 (0.011, 0.046)	0.003
**Less Prescribed DMTs**							
**alemtuzumab**	2011-2022	NA	NA	-0.011 (-0.028, 0.007)	0.207	-0.011 (-0.028, 0.007)	0.207
**cladribine**	2006-2022	I-NA	2019	0.039 (0.024, 0.055)	<0.001	-0.106 (-0.261, 0.048)	0.159
**daclizumab**	2002-2018	NA	NA	-0.012 (-0.025, 0.001)	0.064	-0.012 (-0.025, 0.001)	0.064
**dioroximel fumarate**	2020-2022	NA	NA	NA	NA	NA	NA
**mitoxantrone**	2000-2020	D	NA	-0.013 (-0.023, -0.003)	0.013	-0.013 (-0.023, -0.003)	0.013
**monomethyl fumarate**	2020-2022	NA	NA	NA	NA	NA	NA
**ofatumumab**	2020-2022	NA	NA	NA	NA	NA	NA
**ozanimod**	2020-2022	NA	NA	NA	NA	NA	NA
**peginterferon beta-1a**	2014-2022	NA	NA	0.008 (-0.012, 0.028)	0.383	0.008 (-0.012, 0.028)	0.383

[1] **Trend**: the pattern of MS-related DMT prescription over time assessed using cosine similarity.
I, increasing throughout;
I-D, increasing trend before the change point, followed by a decreasing trend;
I-I, increase throughout, at different rate before and after the change point;
I-NA, increasing trend with no significant trend after the change point;
D, decreasing throughout;
D-I, decreasing trend, followed by an increasing trend;
D-NA, decreasing trend with no significant trend after the change point;
NA-D, no significant trend before the change point, followed by a decreasing trend;
NA-I, no significant trend before the change point, followed by an increasing trend;
NA, insufficient information to estimate the temporal trend.

[2] **Change point**: the year during which a statistically significant shift in the MS-related DMT prescription trend occurred.
