# Supplementary material for: Knowledge graph-guided multiple sclerosis identification and therapeutic trend analysis: Real-world evidence from two large healthcare systems
Source: PLOS Digit Health. 2026 Aug 28;5(8):e0001554. doi: 10.1371/journal.pdig.0001554 (PMC13524337; doi:10.1371/journal.pdig.0001554)
Supplement: Supplementary file 4 [file pdig.0001554.s004.pdf]

S4 Table. Performance of the final KOMAP MS phenotyping algorithm against a benchmark algorithm.

The performance metrics of the KOMAP phenotyping algorithm using codified features plus narrative (CUI, concept unique identifier) features at UPMC and MGB were shown along with a rule-based benchmark algorithm using claims data at KPSC as reported by Culpepper et al.[3]

	**KPSC (Algorithm E)**	**UPMC (KOMAP: codified + CUI)**	**MGB (KOMAP: codified + CUI)**
**Algorithm Definition**	≥3 MSrelated claims (IP/OP/DMT) within 2 years.	KOMAP with both structured features plus CUIs	KOMAP with both structured features plus CUIs
**AUROC**	NA	0.922	0.994
**Accuracy**	85.90%	85.70%	95.30%
**Sensitivity**	0.881	0.794	1.000
**Specificity**	0.709	0.900	0.900
**PPV**	0.954	0.953	0.764
**NPV**	0.466	0.634	1.000

AUROC, area under the receiver operating characteristics curve; *DMT*, disease-modifying therapy; *IP*, inpatient; *OP*, outpatient; *NPV*, negative predictive value; *PPV*, positive predictive value.
