# Supplementary material for: Knowledge graph-guided multiple sclerosis identification and therapeutic trend analysis: Real-world evidence from two large healthcare systems
Source: PLOS Digit Health. 2026 Aug 28;5(8):e0001554. doi: 10.1371/journal.pdig.0001554 (PMC13524337; doi:10.1371/journal.pdig.0001554)

S1 Fig. Trends of commonly prescribed MS-related DMTs (2004-2022)

A. dimethyl fumarate, **B.** fingolimod, **C.** glatiramer acetate, **D.** interferon beta-1a, **E.** interferon beta-1b, **F.** natalizumab, **G.** ocrelizumab, **H.** rituximab, **I.** terifluonomide, and **J.** methylprednisolone (included as a reference). For each DMT, the top panel displays the dynamic cosine similarity with 95% confidence intervals. The bottom panel shows the DMT frequency, defined as the proportion of MS patients prescribed the target drug in each calendar year.

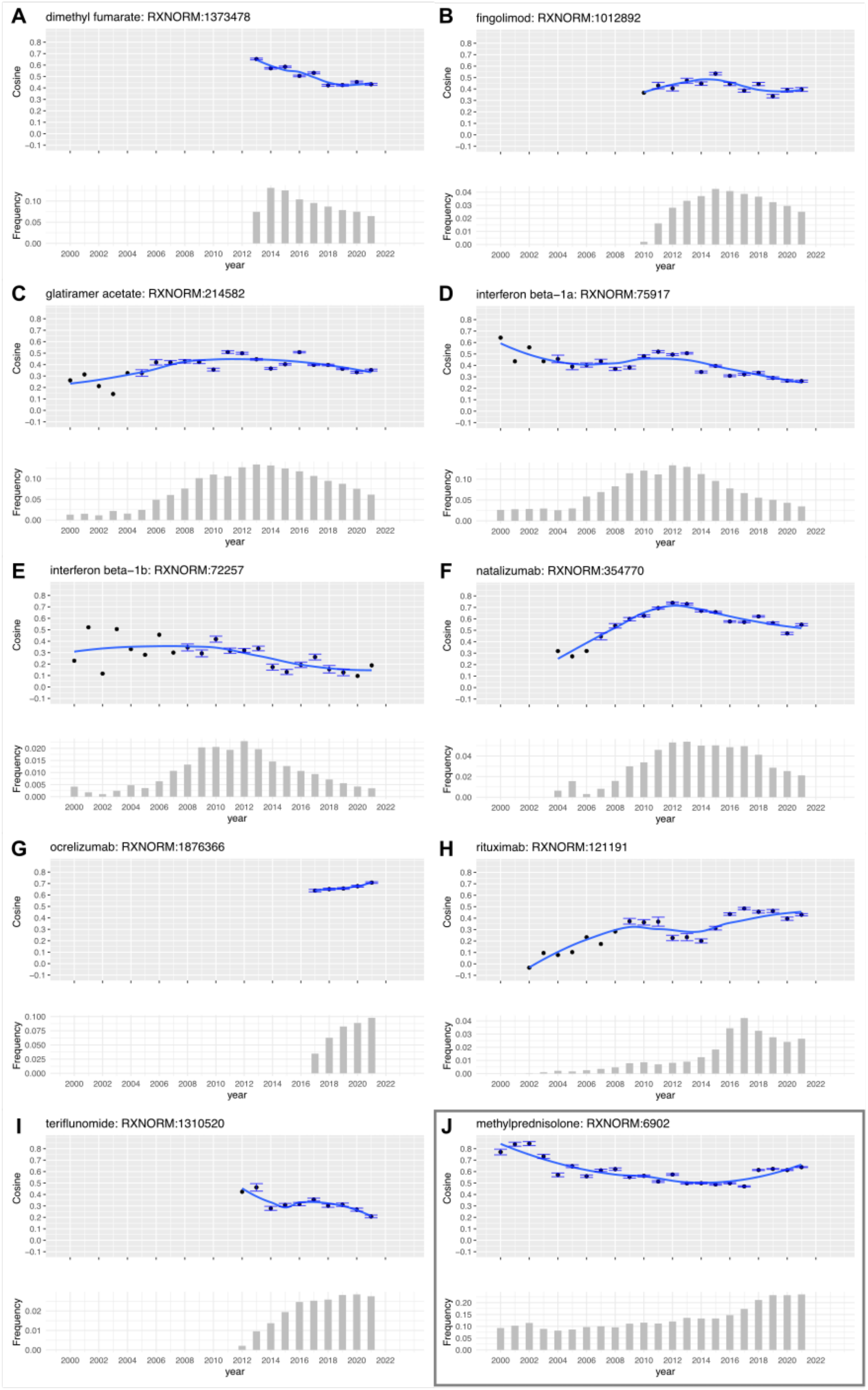

Supplement: Supplementary file 5 [file pdig.0001554.s005.pdf]
