# Supplementary material for: Knowledge graph-guided multiple sclerosis identification and therapeutic trend analysis: Real-world evidence from two large healthcare systems
Source: PLOS Digit Health. 2026 Aug 28;5(8):e0001554. doi: 10.1371/journal.pdig.0001554 (PMC13524337; doi:10.1371/journal.pdig.0001554)

S2 Fig. Trends of less commonly prescribed MS-related DMTs (2004 and 2022).
A. alemtuzumab, **B.** cladribine, (**C.** daclizumab (withdrawn), **D.** dioroximel fumarate, **E.** mitoxantrone, **F.** monomethyl fumarate, **G.** ofatumumab, **H.** ozanimod, **I.** peginterferon beta-1a and **J.** siponimod. For each DMT, the top panel displays the dynamic cosine similarity with 95% confidence intervals. The bottom panel shows the DMT frequency, defined as the proportion of MS patients prescribed the target drug in each calendar year.

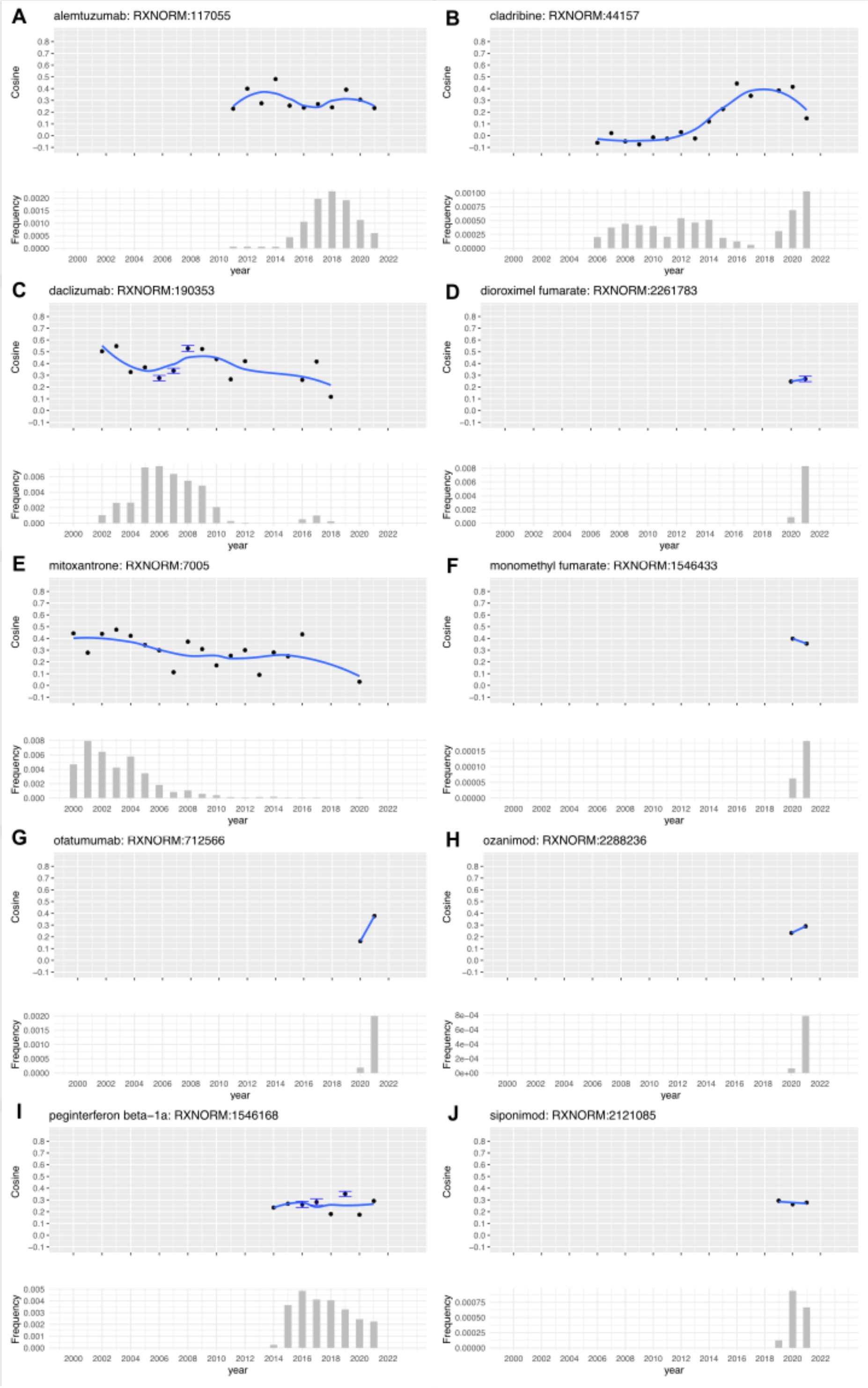

Supplement: Supplementary file 6 [file pdig.0001554.s006.pdf]
