# Supplementary material for: Knowledge graph-guided multiple sclerosis identification and therapeutic trend analysis: Real-world evidence from two large healthcare systems
Source: PLOS Digit Health. 2026 Aug 28;5(8):e0001554. doi: 10.1371/journal.pdig.0001554 (PMC13524337; doi:10.1371/journal.pdig.0001554)

S3 Fig. Sensitivity of Temporal MS-DMT Cosine Similarity Trends to Alternative LOESS Smoothing Parameters (2004-2022).

A. alpha4-integrin blockers; **B.** glatiramer acetate; **C.** B-cell depletion therapies; **D.** interferon-beta. For each representative DMT mechanism class, LOESS curves are fitted using span parameters of 0.3 (purple), 0.5 (blue), and 0.7 (green). 0.5 is the primary specification, whereas 0.3 and 0.7 are the alternative LOESS span values.

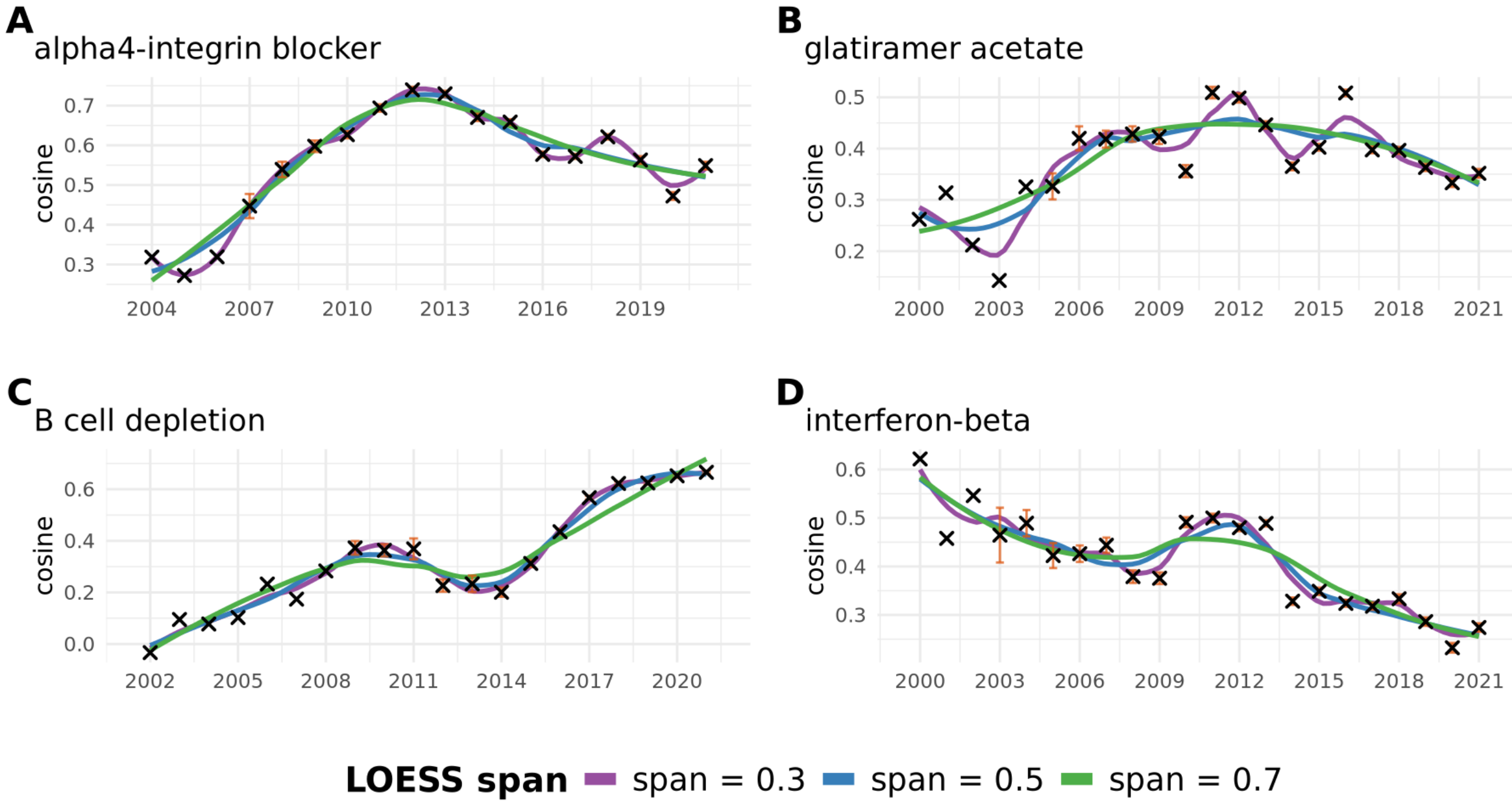

Supplement: Supplementary file 7 [file pdig.0001554.s007.pdf]
